# Alterations of lipid homeostasis in morbid obese patients are partly reversed by bariatric surgery

**DOI:** 10.1016/j.isci.2024.110820

**Published:** 2024-08-26

**Authors:** Flore Sinturel, Simona Chera, Marie-Claude Brulhart-Meynet, Jonathan Paz Montoya, Etienne Lefai, François R. Jornayvaz, Giovanni D’Angelo, Minoa Karin Jung, Zoltan Pataky, Howard Riezman, Charna Dibner

**Affiliations:** 1 Division of Thoracic and Endocrine Surgery, Department of Surgery, University Hospitals of Geneva, 1211 Geneva, Switzerland; 2 Department of Cell Physiology and Metabolism, Faculty of Medicine, University of Geneva, 1211 Geneva, Switzerland; 3 Diabetes Center, Faculty of Medicine, University of Geneva, 1211 Geneva, Switzerland; 4 Institute of Genetics and Genomics in Geneva (iGE3), 1211 Geneva, Switzerland; 5 Department of Clinical Science, University of Bergen, Bergen, Norway; 6 Proteomics Core Facility, EPFL, 1015 Lausanne, Switzerland; 7 Institute of Bioengineering, School of Life Sciences, EPFL, Lausanne, Switzerland; 8 INRA, Unité de Nutrition Humaine, Université Clermont Auvergne, Paris, France; 9 Division of Endocrinology, Diabetes, Nutrition, and Therapeutic Patient Education, Unit of therapeutic patient education, WHO Collaborating Centre, Department of Medicine, University Hospital of Geneva, 1211 Geneva, Switzerland; 10 Division of Visceral Surgery, Department of Surgery, University Hospital of Geneva, 1211 Geneva, Switzerland; 11 Department of Biochemistry, Faculty of Science, NCCR Chemical Biology, University of Geneva, 1211 Geneva, Switzerland

**Keywords:** Health sciences, Medicine, Lipidomics

## Abstract

Besides its beneficial effect on weight loss, gastric bypass surgery (GBS) may impact the circulating levels of phospho- and sphingolipids. However, long-term effects have not been explored.

To investigate alterations in lipidomic signatures associated with massive weight loss following GBS, we conducted direct infusion tandem mass spectrometry on serum and subcutaneous adipose tissue (SAT) samples collected in a longitudinal cohort of morbid obese patients prior to GBS and 1 year following the surgery.

A tissue-specific rearrangement of 13% among over 400 phospholipid and sphingolipid species quantified in serum and SAT was observed 1 year following GBS, with a substantial reduction of ceramide levels and increased amount of hexosylceramides detected in both tissues. The comparison of these new lipidomic profiles with the serum and SAT lipidomes established from an independent cohort of lean and morbid obese subjects revealed that GBS partly restored the lipid alterations associated with morbid obesity.

## Highlights

• GBS results in phospho- and sphingolipid rearrangement in morbid obese patients
• GBS impacts differentially the lipid metabolism in serum and SAT
• GBS partly restores the lipid landscape associated with morbid obesity in humans
• Long-term adaptations of lipid homeostasis concomitant with post-GBS weight loss

## Graphical abstract

**Figure:**
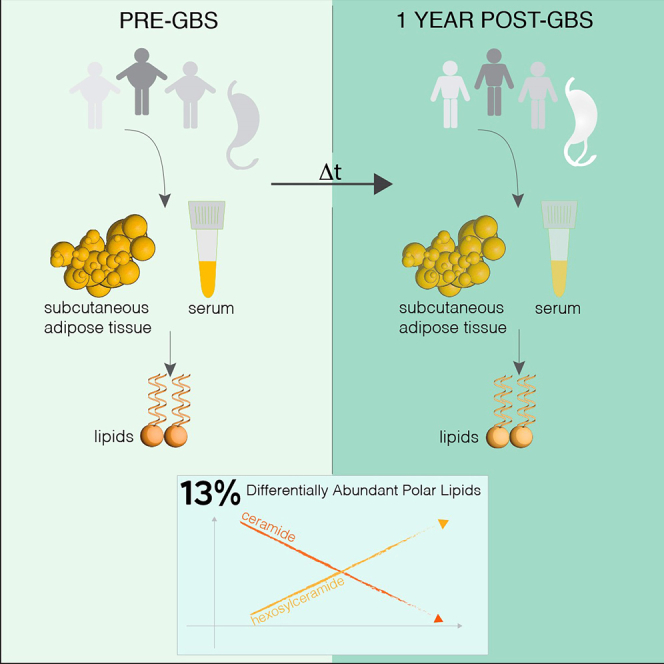

## Introduction

The Roux-en-Y gastric bypass surgery (GBS) is a commonly used treatment applied to counteract morbid obesity in modern countries. It usually leads to a drastic and fast reduction of weight and fat mass. Whereas weight loss and glucose metabolism improvement following GBS have been well documented,^1^^,^^2^^,^^3^ long-term modifications of lipid metabolism following GBS stay largely unexplored. Indeed, all the studies conducted so far on the impact of the surgery-induced weight loss on the human plasma lipidome were performed 1–3 months following the surgery.^4^^,^^5^^,^^6^^,^^7^^,^^8^ Moreover, such metabolomic or lipidomic studies focused on a limited range of lipid metabolites in the serum and did not examine the full spectrum of GBS-induced lipid changes. With the recent advances in lipidomics approaches, a broad range of individual phospho- and sphingolipid species can be at present quantified in human plasma and metabolic tissues, greatly expanding our knowledge regarding the complexity of lipid landscape dysregulation in metabolic diseases.^9^^,^^10^^,^^11^^,^^12^^,^^13^

We therefore established the systematic lipid signatures in a longitudinal cohort of morbidly obese subjects, conducted prior to and 1 year after GBS (cohort 1). To this end, lipidomic approach based on direct infusion-tandem mass spectrometry (MS) has been employed, allowing to quantify a wide spectrum of phospho- and sphingolipid metabolites. We provide for the first time a detailed analysis of the alterations of lipid homeostasis occurring in the subcutaneous adipose tissue (SAT) collected from the same individuals in parallel to the blood samples, prior to GBS and 1 year later. We completed these novel analyses by cross-comparing the data from cohort 1 with our previous lipidomic analyses conducted by the same approach in a cohort of morbid obese patients in serum and visceral adipose tissue (VAT) samples (cohort 2).

## Results

### Alterations of clinical and metabolic features in morbid obese subjects 1 year following GBS as compared to the status prior to surgery (cohort 1)

Table S1 reports the comparison of the main anthropometrical and serum profile characteristics of 11 morbid obese patients (cohort 1) before they underwent the surgery (pre-GBS) and approximately 1 year after the intervention (post-GBS).^14^ As previously described, the GBS induced a significant reduction of the total body weight, fat mass, waist, and hip circumferences, as compared to baseline morbid obese (pre-GBS) (Table S1^14^). In addition, 1 year post-GBS serum profiles exhibited a significant reduction of total blood cholesterol, LDL cholesterol (LDL-c), fasting insulin and leptin levels, as well as levels of several inflammatory biomarkers (Table S1).

### Substantial alterations of the sphingolipid metabolism in serum following GBS (cohort 1)

**Figure 1:**
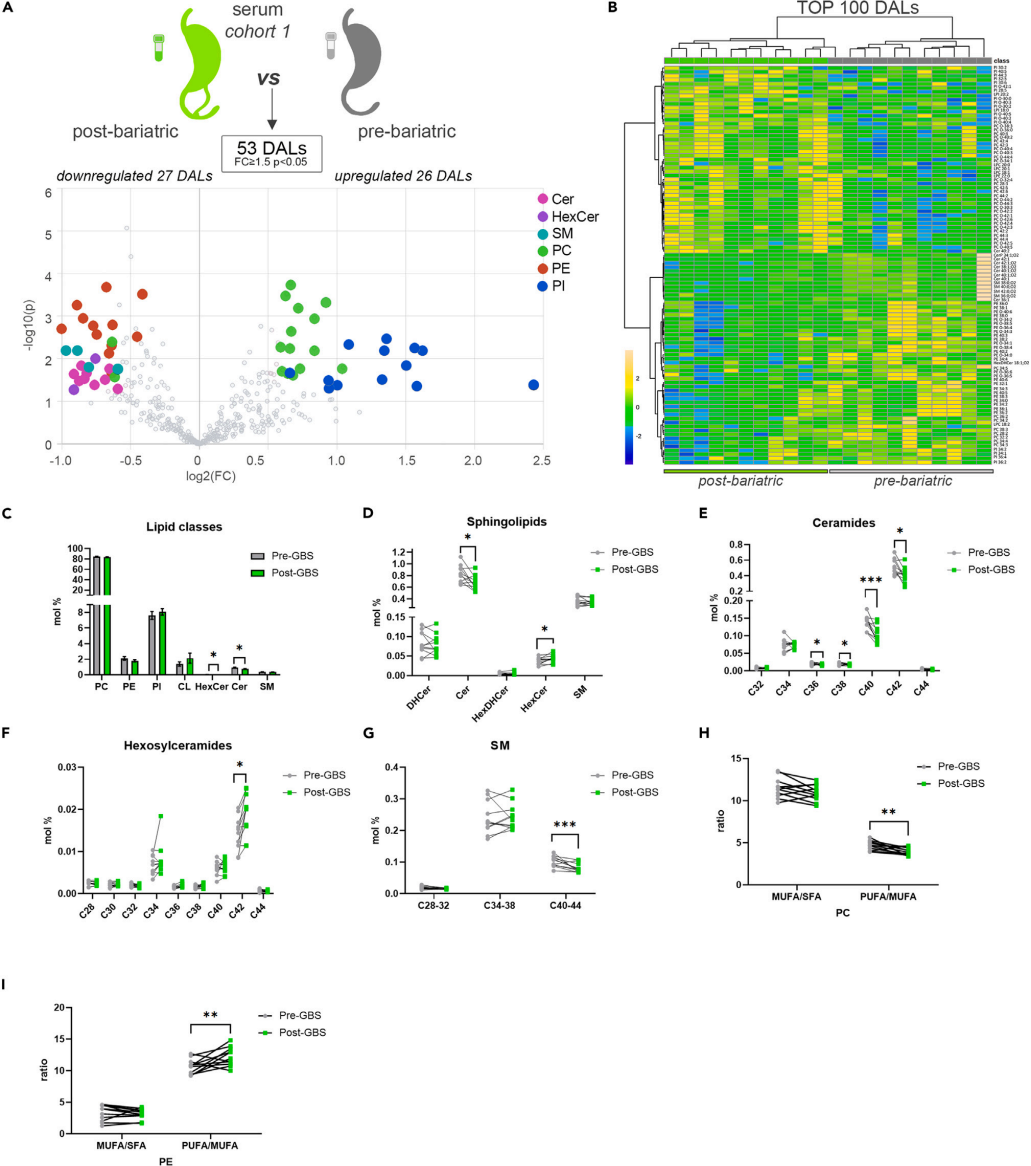
Comparison of differentially abundant serum lipids measured in morbid obese individuals before and after bariatric surgery (*cohort 1*) (A) Volcano plot of the DALs between pre- and post-bariatric serum samples (cohort 1, n = 11). Colored dots highlight significant up- or down-regulated individual lipid species (fold change ≥1.5 and p < 0.05, paired design). (B) Hierarchical analysis *(Distance Measure: Euclidian; Clustering algorithm: Ward)* of top 100 serum lipids with most contrasting patterns between pre- and post-bariatric serum samples (n = 11). (C) Lipid class repartition (PC, PE, PI, CL, HexCer, Cer, and SM) in human serum collected before and after GBS (in mol %). (D) Relative level changes (mol %) of DHCer, Cer, HexDHCer, and HexCer in sera collected before and after GBS. (E and F) Relative Cer (E) and HexCer (F) level changes (mol %) in serum collected before and after GBS, represented according to the chain length. (G) Relative SM level changes (mol %) in sera collected before and after GBS, represented according to the chain length. (H and I) PC (H) and PE (I) ratios of MUFA to SFA and of PUFA to MUFA detected in sera collected before and after GBS. Statistics for (C–I) are paired t test. Data are represented as mean ± SEM. ∗p < 0.05; ∗∗p < 0.01; ∗∗∗p < 0.001. See also Figure S1.

Employing MS approach, we established detailed lipidomic signatures in serum samples collected before and 1 year after GBS from the eleven patients (*n* = 22 paired samples**)**. In parallel, 5 among these 11 patients were subjected to SAT biopsy collection before and 1 year after GBS (*n* = 10 paired samples). Our lipidomic analyses detected a total of 400 polar lipid metabolites in sera (Table S2) and 413 in SAT samples (Table S3) across all the subjects. The lipidomics performed on the serum samples collected before and after the surgery from the same patients revealed 53 lipid metabolites that were significantly differentially abundant (FC > 1.5; *p* < 0.05) (Figure 1A). When we performed hierarchical clustering on 100 top differentially abundant lipids (DALs), not all of which were qualified as significantly different, a clear separation of the samples collected prior to and 1 year after the GBS (Figure 1B) was observed. Two classes of sphingolipids, hexosylceramides (HexCer) and ceramides (Cer), exhibited the most striking changes in the patient sera over a year’s time post-GBS (Figures 1A–1D). Decrease in Cer lipids was concomitant with an increase in the HexCer species after GBS (Figures 1D–1F). Interestingly, the changes observed in mature sphingolipids (dihydroceramides, DHCer) were not paralleled with a similar modification of the precursor sphingolipid forms (hexosyldihydroceramides, HexDHCer) (Figure 1D), suggesting that the decrease in Cer levels may not stem from a downregulation of the *de novo* synthesis. The most striking decrease in Cer was observed for the most abundant species: Cer40 and Cer42 (Figure 1E). In parallel, the level of the most abundant HexCer metabolite HexCer C42 was significantly increased in the samples collected after GBS (Figure 1F). Although the total amount of SM was not modified after GBS (Figure 1C), the levels of the long-chain SM species were significantly reduced (Figure 1G). Overall, 20% of the detected sphingolipids were significantly decreased in the samples collected after GBS, suggesting a massive rearrangement of sphingolipid metabolism during the 1-year time following GBS.

Furthermore, both the hierarchical clustering and volcano plot analyses revealed significant changes in the levels of several serum phosphatidylcholine (PC), phosphatidylinositol (PI), and phosphatidylethanolamine (PE) species following GBS (Figures 1A and 1B). Among the PC lipid species that exhibited altered levels post-GBS, we noticed an increase in very long chain (VLC) PCs (Figure S1A). Additionally, a decrease in the MUFA/PUFA PC ratio has been observed due to a significant decrease in PUFA species, along with an increase of SFA and MUFA PC species (Figures 1H, S1B, and S1C). By contrast, the MUFA/PUFA ratio was increased, whereas the SFA decreased within the PE group (Figures 1I and S1D). The ether PE species were significantly downregulated in the patient sera following GBS surgery as compared to their levels in serum samples collected prior to the surgery (Figure S1E).

### In human SAT, PI and HexCer metabolite levels are increased following GBS (cohort 1)

**Figure 2:**
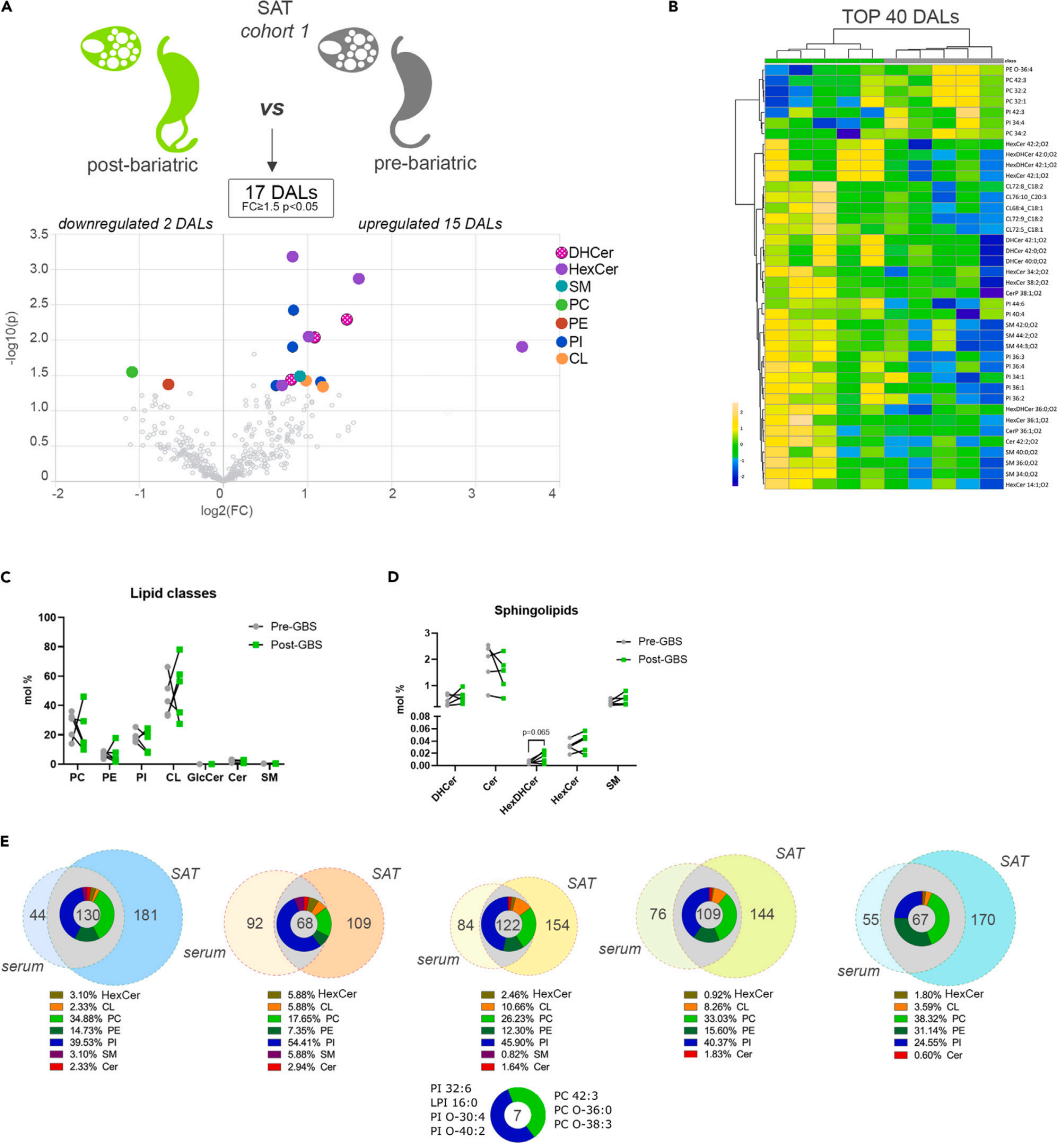
Comparison of differentially abundant SAT lipids measured in morbid obese individuals before and after bariatric surgery (*cohort 1*) (A) Volcano plot of the differentially abundant lipids between pre- and post-bariatric SAT samples (*n* = 5). Colored dots highlight significant up- or down-regulated individual lipid species (fold change ≥1.5 and *p* < 0.05, paired design). (B) Hierarchical analysis *(Distance Measure: Euclidian; Clustering algorithm: Ward)* of top 40 SAT lipids with most contrasting patterns between pre- and post-bariatric SAT samples (*n* = 5). (C) Lipid class repartition (PC, PE, PI, CL, HexCer, Cer, and SM) in human SAT collected before and after GBS (in mol %). (D) Relative level changes (mol %) of DHCer, Cer, HexDHCer, and HexCer in human SAT collected before and after GBS. (E) Venn diagrams assessing the overlap between SAT and serum DALs (fold change ≥1.5 and *p* < 0.05) in each subject following GBS. Pie diagrams depict the range of lipid classes regulated in both SAT and serum following GBS. Seven lipids were differently regulated in both SAT and serum across all five analyzed individuals. Statistics for (C and D) are paired t test. Data are represented as mean ± SEM. See also Figure S2.

In the SAT samples, overall changes in the measured lipid levels were milder than those observed in the sera from the same individuals (Figures 2A and 2B). Indeed, the total amount of lipids clustered by lipid class did not significantly differ (Figure 2C). Volcano plot analysis revealed that 17 individual lipids were significantly altered, and the top 40 DALs showed a distinct separation between the sample groups (Figures 2A and 2B). The upregulated lipid metabolites were mainly represented by PI and sphingolipids metabolites, in particular by the HexCer (Figures 2A and 2B). Indeed, we observed an increase in these lipid species, as well as HexDHCer, although the difference in their total amount did not reach statistical significance (Figure 2D). The total amount of the Cer was unchanged in *n* = 2 individuals, whereas it was decreased in three others post-GBS (Figure 2D). By contrast, three DHCer species (DHCer42:1; O_2_, DHCer42:0; O_2_, and DHCer40:0; O_2_) were significantly more abundant in SAT samples collected after GBS as compared to baseline (Figures 2A and 2B). Several long-chain (LC) SM species were increased as well, resulting in an opposite trend compared to the one that was observed in the serum (Figure S2A).

### Increased levels of PI metabolites 1 year following GBS in both serum and SAT of morbid obese patients (cohort 1)

Comparison between the lipid composition of serum and SAT revealed a clear difference in the lipid class repartition between the two. Indeed, CLs were predominantly observed in SAT, whereas PC lipids were mainly found in serum (Figures 1C and 2C). In spite of such tissue specificity of lipid composition, a substantial overlap between DALs was observed in both serum and SAT for all the five subjects where we analyzed both tissues (Figure 2E). In addition, seven lipids (three PC and four PI metabolites) were differentially regulated across all donors and tissues (Figure 2E). Moreover, although the PI constituted only 8% to 20% of all the lipids detected in both serum and SAT (Figures 1C and 2C), they represented, on average, more than 40% of the serum and SAT overlapping DALs (Figure 2E), revealing that PI lipid metabolites overcame the most pronounced alterations in both serum and SAT following GBS.

### Massive alteration of the serum and visceral adipose tissue lipid landscape upon morbid obesity (cohort 2)

**Figure 3:**
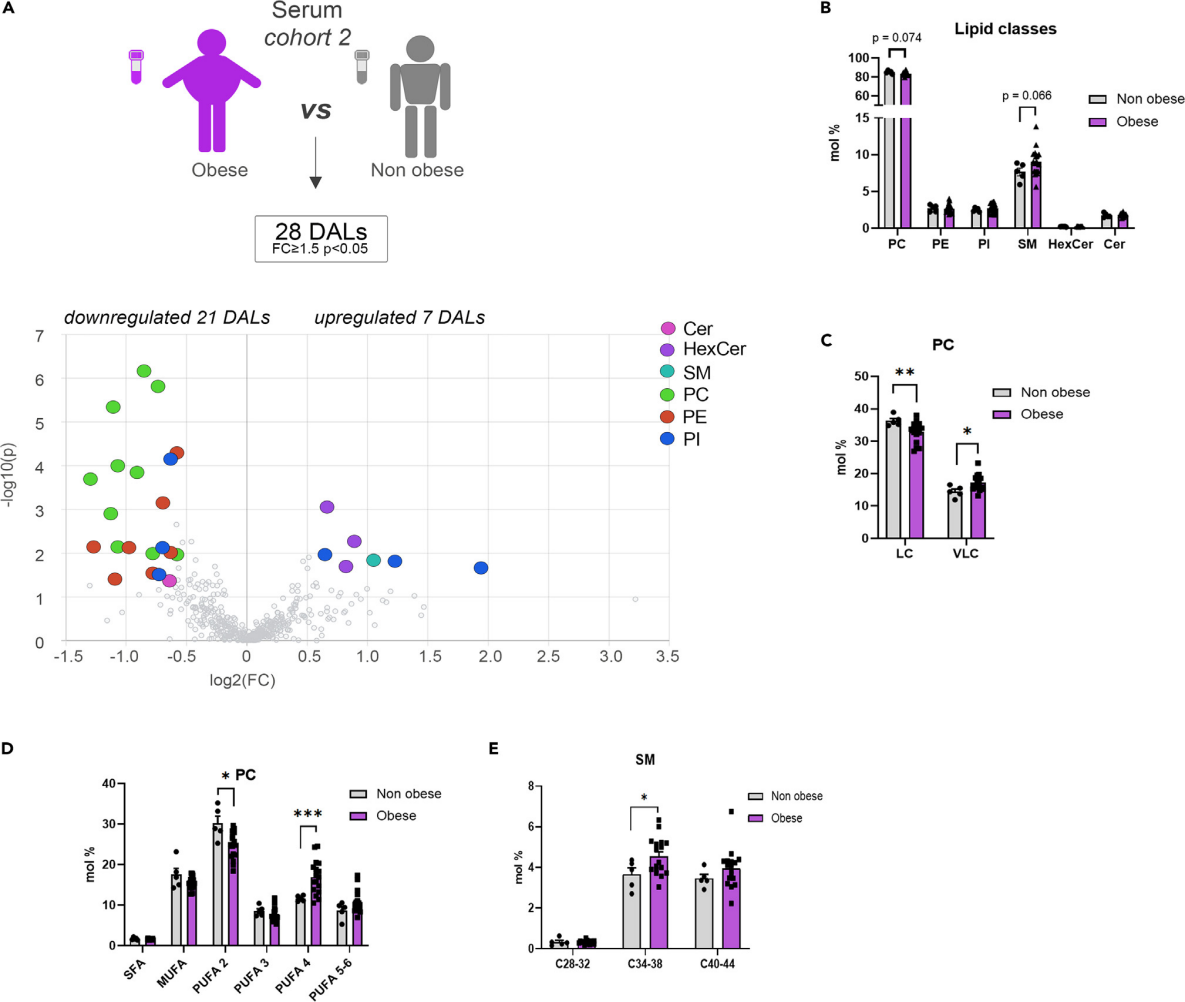
Serum lipids measured in morbid obese and lean control individuals (*cohort 2*) (A) Volcano plot of the differentially abundant lipids in serum between obese and lean control subjects. Colored dots highlight significant up- or down-regulated individual lipid species (fold change ≥1.5 and *p* < 0.05, Welch’s corrected). (B) Lipid class repartition (PC, PE, PI, CL, HexCer, Cer, and SM) in human serum from lean control and obese individuals (in mol %). (C) Relative PC level changes (mol %) in serum collected from lean control and obese individuals, clustered according to the chain length, LC (long chain C28-34), VLC (very long chain C38-44). (D) Relative PC level changes (mol %) in serum collected from lean control and obese individuals, represented according to the degree of saturation. (E) Relative SM level changes (mol %) in serum collected from lean control and obese individuals, clustered according to the chain length. Statistics for (B–E) are unpaired Student’s t test *p* values. Data from obese (*n* = 16) and control (*n* = 5) individuals are represented as mean ± SEM. ∗*p* < 0.05, ∗∗*p* < 0.01; ∗∗∗*p* < 0.001.

**Figure 4:**
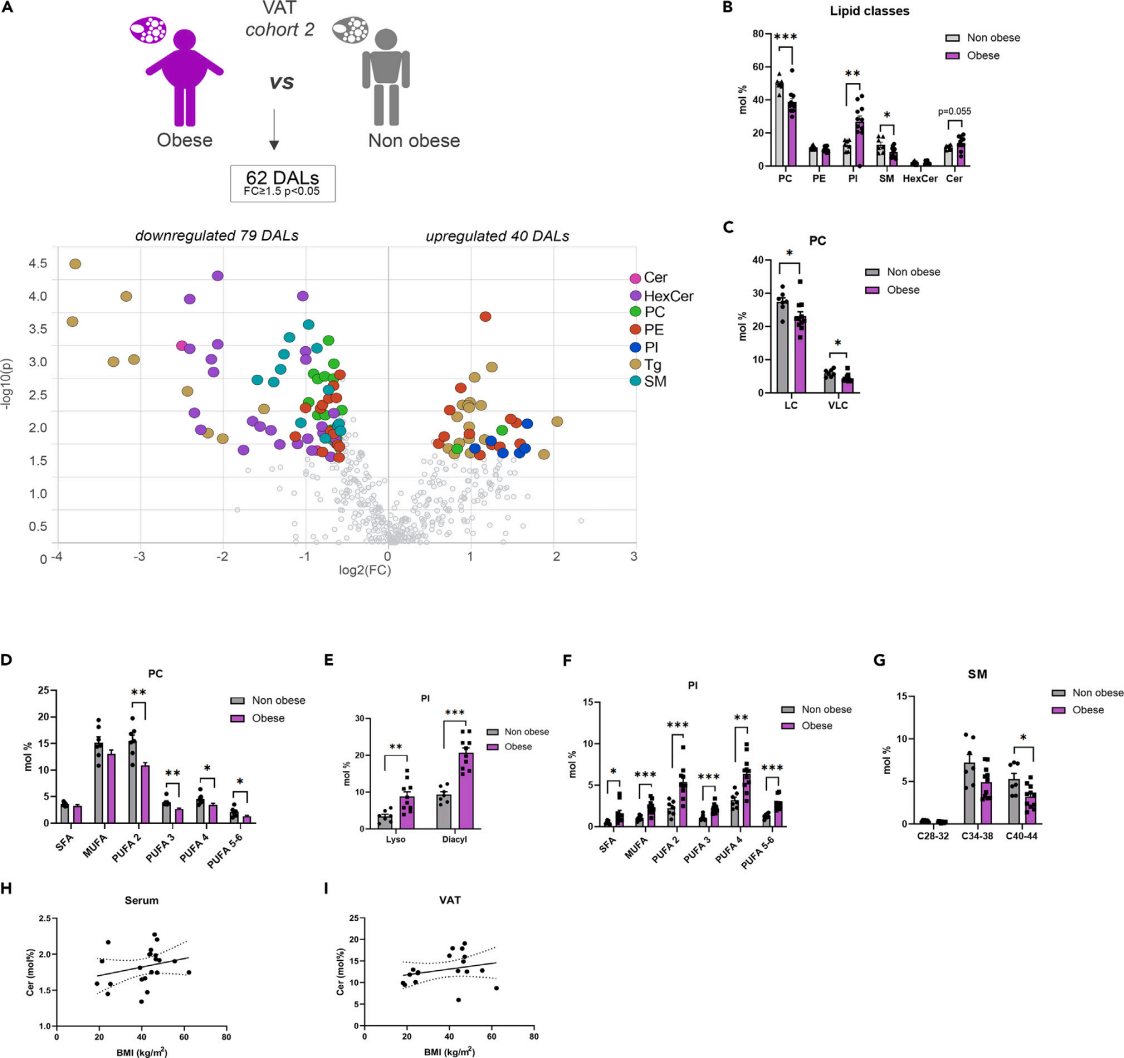
VAT lipids measured in morbid obese and lean control individuals (*cohort 2*) (A) Volcano plot of the differentially abundant lipids in VAT between obese and lean control subjects. In addition to the lipid classes measured in the other samples, triglycerides (Tg) were measured. Colored dots highlight significant up- or down-regulated individual lipid species (fold change ≥1.5 and *p* < 0.05, Welch’s corrected). (B) Lipid class repartition (PC, PE, PI, CL, HexCer, Cer, and SM) in human VAT from lean control and obese individuals (in mol %). (C and D) Relative PC level changes (mol %) in VAT collected from lean control and obese individuals, clustered according to the chain length (C), and represented according to the degree of saturation (D). LC (long Chain C28-34), VLC (Very Long Chain C38-44). (E and F) Relative PI level changes (mol %) in VAT, represented according to the nature of the fatty acid linkage [diacyl or monoacyl (lyso)] (E) and the degree of saturation (F). (G) Relative SM level changes (mol %) in VAT collected from lean control and obese individuals, clustered according to the chain length. (H and I) Association between the relative levels of ceramides detected in sera (H) or VAT (I), and the BMI of the subjects (serum *n* = 21, VAT *n* = 17, Spearman correlation, for the serum R = 0.412, *p* = 0.057, for the VAT R = 0.315, *p* = 0.2). Statistics for (B–G) are unpaired Student’s t test *p* values. Data from obese (*n* = 11) and control (*n* = 7) individuals are represented as mean ± SEM. ∗*p* < 0.05, ∗∗*p* < 0.01; ∗∗∗*p* < 0.001.

In order to complete our investigation of the GBS effects on the lipidomic features of morbid obese subjects, we reanalyzed the serum and VAT lipidomes conducted recently,^9^ in a sub-cohort of lean and morbid obese individuals (cohort 2) (Figures 3 and 4 and Table S4). In sera, a number of individual PC lipids exhibited decreased levels in morbid obese individuals compared to the control samples (Figure 3A), although no significant difference was observed for the overall PC lipid class alterations (Figure 3B). Strikingly, while the long-chain (LC) PC metabolite levels were significantly decreased in serum of morbid obese patients, the levels of VLC PCs were significantly increased (Figure 3C). Similarly, we observed a diminution of the PUFA 2 PC lipids in morbid obesity, paralleled with upregulation of PUFA 4 (Figure 3D). Additionally, the sera obtained from obese individuals contained increased SM lipid levels (Figure 3B), in particular C34-38 SM lipids (Figure 3E).

Noteworthy, the most striking alterations in the polar lipid metabolism upon morbid obesity were observed in VAT. Indeed, morbid obese individuals exhibited decreased levels of PC lipids (Figures 4A–4D), similarly to the serum, associated with increased levels of PI metabolites compared to their control counterparts (Figures 4A, 4B, 4E, and 4F). All the PC lipids were decreased, irrespective of the chain length or the degree of saturation of the species (Figures 4C and 4D). In contrast, the lyso- and diacyl PIs at all levels of saturation were overrepresented in VAT of morbid obese individuals (Figures 4E and 4F). In addition, there is an unambiguous diminution of SM lipids and many HexCer metabolites in obese individuals compared to their control counterparts (Figures 4A, 4B, and 4G). By contrast, Cer levels were elevated in the VAT of morbid obese individuals (Figure 4B).

### Bariatric surgery partly restores the lipid metabolism imbalance observed upon morbid obesity

A comparison between the lipid profiles observed in the sera and VAT of the cohort 2 subjects (Figures 3 and 4) and those obtained in pre- and post-GBS patient samples from the cohort 1 (Figures 1 and 2) suggests that the GBS partially rescues the lipidome landscape changes associated with obesity. Indeed, we observed a clear inversion of the sphingolipid phenotype associated with obesity, which was characterized by lower levels of SM and HexCer lipids (Figures 4A, 4B, and 4G), in the patients following GBS (Figures 2A–2D). Although the difference in Cer levels in VAT derived from obese subjects compared to the control counterpart did not reach statistical significance (Figure 4B), we observed a trend for an association between the patient BMI and the percentage of Cer measured in serum and VAT (Figures 4H and 4I). To a lesser extent, this correlation was conserved in our cohort of obese individuals who underwent GBS (Figure S2B), while the levels of HexCer were inversely associated with the BMI (Figure S2C). This result suggests that the accumulation of Cer during the weight gain can be reversed by GBS, possibly via a conversion of Cer into HexCer. Concerning the glycerophospholipids, no clear inversion of the obese phenotype upon GBS has been observed. Indeed, high levels of VLC PC were detected in sera from morbid obese subjects (cohort 2; Figure 3C), and morbid obese patients 1 year after GBS (cohort 1; Figure S1A). Similarly, the weight loss induced by the surgery was not sufficient to counteract the PI accumulation in SAT that characterized morbidly obese subjects (Figure 4B), with the high abundance of some PI species still observed 1 year following GBS (cohort 1; Figures 1A, 1B, 2A, and 2B).

## Discussion

Our study provides the first in-depth lipidome profiling of dynamic changes of the lipid homeostasis in serum and SAT from morbid obese patients 1 year following GBS. The spectrum of DALs clearly differed between pre- and post-GBS samples and was not identical in serum and SAT, highlighting lipid-tissue-specific rearrangement upon GBS. Furthermore, the comparison of the lipidomic profiles from the morbid obese patients who overcame GBS (cohort 1) with our recent lipidomic analyses conducted in an independent cohort of morbid obese and lean control individuals (cohort 2) suggests that GBS partially rescues the alterations of lipid homeostasis associated with morbid obesity in humans (Table S5).

So far, most of the studies reporting lipid changes upon GBS were conducted only in serum samples and used targeted detection methods that cover a limited range of “classic” lipid species.^5^^,^^6^^,^^7^^,^^15^ Here, we present the first in-depth characterization of the phospho- and sphingolipid profile of the serum and SAT of morbid obese human patients who overcame the GBS. Importantly, in contrast to previous works conducted early after GBS,^5^^,^^6^^,^^8^ we aimed at analyzing sustained lipid metabolite changes during a longer period of 1 year following the surgery. Thus, the resulting lipidomic profiles reflect not only the impact of the rapid weight loss but also the long-term metabolic adaptations that were associated to this change in serum and in SAT. Our data support some of the previously proposed serum lipids as hallmark of weight loss following GBS. Thus, we confirmed a substantial decrease in serum Cer^5^^,^^6^^,^^8^^,^^15^ and LC SM^4^^,^^6^ species upon GBS (Figures 1C–1E and 1G). Strikingly, our data demonstrate, for the first time, that the substantial alterations in the lipid landscape in morbid obese patients following GBS comprise the increased levels of HexCer (Figures 1D and 1F). Altogether, our findings suggest that 1 year after the GBS, sphingolipid landscape shifts from Cer toward HexCer. Concerning the phospholipids, our results contrast with some recent works focused on serum lipid metabolic changes early after GBS. Indeed, although we consistently reported a significant decrease of ether PE in the serum 1 year following the surgery (Figure S1E),^8^ we did not observe anymore a widespread decrease in PCs and/or PIs^4^^,^^6^^,^^8^ in this context. This suggests that the diminution in these lipid classes is primarily associated with acute weight loss rather than reflecting a long-term effect, although we cannot formally rule out that these differences are related to methodological discrepancies between our study and previous works.

One strength of the present study is the comparison of the lipidomic data obtained from samples collected upon GBS, with our recent lipidomic analyses of the lipid alterations associated with morbid obesity performed in an independent human cohort.^9^ In the sera of morbid obese individuals, the levels of two choline-containing phospholipid classes were drastically changed: an overall decrease in PCs was concomitant with an increase in SMs (Figures 3A–3E). This observation agrees with previous studies showing that SMs accumulate in the serum of patients suffering from severe obesity and insulin resistance.^16^^,^^17^^,^^18^ Similarly, the levels of several PC species in the serum were recently found to be inversely associated with obesity.^10^ Strikingly, these obesity-related changes in the lipid metabolites were partly reversed in the patients 1 year following GBS (Table S5). Of note, as SMs are major components of LDL and VLDL particles,^19^ the lower abundance of serum SMs species post-GBS may reflect the significant reduction of plasma concentrations of LDL (Table S1). Additional substantial change observed in post-GBS samples was the reduction of Cer levels, concomitant with increased HexCer amounts in serum and to a lesser extent in SAT (Figures 1A–1F and 2A–2D). Cer is well known to accumulate during obesity, with decreased Cer levels being associated with increase in insulin sensitivity in mice and humans.^20^^,^^21^^,^^22^^,^^23^ In this line, we hereby report that Cer lipids are more abundant in the VAT of morbid obese individuals as compared to lean counterparts (Figures 4A and 4B). By contrast, we observed a significant upregulation of three DHCer species in SAT after the surgery (Figures 2A and 2B). The MS analyses that we employed here cannot distinguish DHCer lipid species from the toxic deoxyceramide (DeoxCer) metabolites that were recently identified in the human adipose tissue.^9^^,^^11^ Indeed, DeoxCer exhibited pronounced accumulation in the VAT of obese T2D individuals as compared to non-T2D obese subjects.^9^ We did measure DeoxCer lipids in the cohort 2 samples, where they were not identified among the DALs between morbid obese and lean individuals (Figure 4A), further promoting the concept that these lipids are predominantly associated with T2D rather than with obesity alone. Further investigations will be required to explore whether DeoxCer lipid content of adipose tissue will be altered by the weight loss following GBS. Importantly, in this study we established the lipid signatures for the two types of white adipose tissue: subcutaneous, analyzed in cohort 1, and visceral in the cohort 2. Recent comparisons of SAT and VAT lipid content suggested depot-specific lipid features.^9^^,^^11^^,^^24^ In agreement with these reports, we observed higher levels of Cer in VAT in comparison to SAT of both lean and obese origin (Figures 2C and 4B). Moreover, our data reveal elevated levels of CL in SAT in comparison to VAT (Figures 2C and 4B), suggesting a more dynamic mitochondrial activity in SAT. Taken together, our data suggest a strong perturbation of the sphingolipid metabolism in morbid obese patients that is partly counteracted by a GBS intervention.

### Limitations of the study

In conclusion, our results demonstrate a massive rearrangement of the human serum and SAT lipidome in morbid obese individuals, in two independent cohorts of patients. Strikingly, we report an improved sphingolipid signature along with a major overhaul of the most abundant glycerophospholipids (PC and PI) following GBS. The comparison of the serum and SAT lipid landscape highlights tissue-specific lipid modifications, although the follow-up studies on a higher number of subjects will be required to confirm this observation. Further examinations are warranted to determine the mechanistic link between long-term clinical benefits associated with surgically induced weight loss and the reported changes of lipid homeostasis.

## Resource availability

### Lead contact

Further information and requests for resources and reagents should be directed to and will be fulfilled by the lead contact, Charna Dibner (Charna.Dibner@unige.ch).

### Materials availability

This study did not generate new unique reagents.

### Data and code availability

• Lipidomics data supporting the findings of this study are available within the Article and supplemental information.
• This paper does not report original code.
• Any additional information required to reanalyze the data reported in this paper is available from the lead contact upon request.

## Acknowledgments

This work was funded by 10.13039/501100001711Swiss National Science Foundation grants 310030_184708/1 and 310030_219187/1, the 10.13039/501100008494Vontobel Foundation, the 10.13039/501100012653Novartis Consumer Health Foundation, EFSD/Novo Nordisk Programme for Diabetes Research in Europe, the 10.13039/501100012654Olga Mayenfisch Foundation, Fondation pour l'innovation sur le cancer et la biologie, Ligue Pulmonaire Genevoise, 10.13039/501100004361Swiss Cancer League KFS-5266-02-2021-R, 10.13039/100007214Velux Foundation, 10.13039/501100006387Leenaards Foundation, the 10.13039/501100017035ISREC Foundation, and the 10.13039/501100008938Gertrude von Meissner Foundation (C.D.) and 10.13039/501100013161Swiss Life Foundation (F.S.).

## Author contributions

F.S. performed lipidomics experiments; F.S. and S.C. analyzed the lipidomic data and performed statistical analyses; M.C.B.M. and J.P.M. assisted with the lipidomic studies; Z.P. conceptualized the cohort 1; Z.P. and M.K.J. provided the samples; E.F. conceptualized the cohort 2 and provided the samples; G.d.A. and H.R. contributed to the lipidomics conceptualization. C.D. designed and coordinated the study; F.S. and C.D. drafted the manuscript. All authors contributed to the manuscript preparation and approved the final version.

## Declaration of interests

The authors declare no competing interests.

## STAR★Methods

### Key resources table

**Table:** 

REAGENT or RESOURCE	SOURCE	IDENTIFIER
**Biological samples**		

Serum and adipose tissue samples from morbid obese subjects before and after GBS intervention	University Hospital of Geneva (Montecucco et al., 2015)^14^	Geneva ethical commission approval number 2017-00133.
Serum and VAT samples from morbid obese non T2D individuals (cohort 2)	Université Clermont Auvergne, France (Hannich et al., 2020)^9^	French Ethical Committee SUD EST IV (Agreement 12/111

**Chemicals, peptides, and recombinant proteins**		

PC 12:0/12:0	Avanti Polar Lipids Inc.	Cat#850335; CAS: 18194-25-7
PE 17:0/14:1	Avanti Polar Lipids Inc.	Cat#LM-1104; CAS: 958763-89-8
PI 17:0/14:1	Avanti Polar Lipids Inc.	Cat#LM-1504; CAS: 1246304-61-9
PS 17:0/14:1	Avanti Polar Lipids Inc.	Cat#LM-1304; CAS: 1036814-91-1
Cer d18:1/17:0	Avanti Polar Lipids Inc.	Cat#860517; CAS: 67492-16-4
SM d18:1/12:0	Avanti Polar Lipids Inc.	Cat#860583; CAS: 474923-21-2
HexCer d18:1/8:0	Avanti Polar Lipids Inc.	Cat#860540; CAS: 111956-47-9
MTBE (methyl-tert-butyl ether)	Sigma Aldrich	Cat#34875; CAS : 1634-04-4
Methylamine solution(33% in absolute ethanol)	Sigma Aldrich	Cat#534102; CAS: 74-89-5
Chloroform	Acros Organics	Cat#326820010; CAS: 67-66-3
Methanol	Acros Organics	Cat#CAS: 67-56-1
n-butanol	Acros Organics	Cat#CAS: 71363
Ammonium molybdate(VI) tetrahydrate	Acros Organics	Cat#CAS: 12054-85-2
Monopotassium phosphate	Sigma Aldrich	Cat#60229; CAS: 7778-77-0
L-ascorbic acid	Sigma Aldrich	Cat#A92902; CAS: 50-81-7
Perchloric acid 70%	Sigma Aldrich	Cat#244252 ; CAS : 7601-90-3
Hexane	ThermoFisher Scientific	CAS: 110-54-3
Methyl acetate	Sigma Aldrich	CAS: 79-20-9
Acetonitrile	Sigma Aldrich	CAS: 75-05-8

**Software and algorithms**		

TSQ Tune 2.6 SP1 QuickQuan™ Software	ThermoFisher Scientific	Catalog number: IQLAAEGABSFAHQMAPT; https://www.thermofisher.com/order/catalog/product/IQLAAEGABSFAHQMAPT
Xcalibur 4.0 QF2 software	ThermoFisher Scientific	Catalog number: OPTON-30965; https://www.thermofisher.com/order/catalog/product/OPTON-30965
LcmsExplorer	EPFL Lausanne Switzerland	http://lipidomes.epfl.ch/
Lipid Data Analyzer	IGB-TUG Graz University	LDA v. 2.6.3.9; https://www.lipidmaps.org/resources/tools/10?task=4.5
MetaboAnalyst 5.0.	McGill University, Canada	https://www.metaboanalyst.ca/MetaboAnalyst/ModuleView.xhtml
Prism Graph Pad 8.0.	Graphpad	https://www.graphpad.com/

**Other**		

Precellys 24 tissue homogenizer	Bertin Instruments	https://www.bertin-instruments.com/product/sample-preparation-homogenizers/precellys24-tissue-homogenizer/precellys24-4/
Zirconium oxide beads CK14	Labgene Scientific SA	Cat#BER20305

### Experimental model and study participant details

#### Study design and patient characteristics

##### Human cohort 1

The clinical characteristics of the individuals included in the cohort 1 have been previously reported by us in.^14^ Samples were obtained from participants with written informed consent. The study was conducted according to the ethical principles for medical research involving human subjects released by the Declaration of Helsinki and had ethics local committee approval. In this work we only included the participants with morbid obesity (defined as BMI ≥ to 40 kg.m^-2^) pre- and post-GBS (see Table S1). The enrolled subjects were non-smokers and exhibited neither arterial hypertension (blood pressure < 140/90 mmHg) nor diabetes *mellitus* (HbA_1c_ ≤ 5.8% (40 mmol/mol)). Sex (four males and seven females) and age (average 38 years old) of the participants are reported in Table S1. All participants underwent an initial screening visit that comprised a physical examination, blood pressure measurements, euglycemic-hyperinsulinemic clamp test, routine blood chemistry, and serum sampling between 8 and 9 AM following over-night fast. SAT biopsies were taken during planned Roux-en-Y Gastric Bypass with a gastric pouch of 20-30 cm^3^ and an alimentary limb of 150 cm and a biliopancreatic limb of 75 cm. One-year post-GBS, all the participants were subjected again to a final physical examination, euglycemic-hyperinsulinemic clamp test, SAT biopsy, routine blood chemistry, and serum sampling at 8-9 AM following overnight fasting.

##### Human cohort 2

The clinical characteristics of the individuals included in the cohort 2 have been reported by us in.^9^ Samples were obtained from all participants with written informed consent. The study conformed to the Declaration of Helsinki and the experimental protocol (‘DIOMEDE’) was approved by the Ethical Committee SUD EST IV (Agreement 12/111) and performed according to the French legislation (Huriet’s law). In this work, we only analysed the serum and VAT samples from lean control and morbid obese non-diabetic donors who had HbA1c levels inferior to 48 mmol/mol, fasting glycemia inferior to 7 mmol/L, and were not diagnosed with type 2 diabetes (T2D) (see Table S4). Sex and age of the participants are reported in Table S4. VAT biopsies from the same participants were taken during planned bariatric surgery. Serum samples were collected between 8 and 10 AM, following over-night fasting.

### Method details

#### Serum and white adipose tissue sample preparation

Blood samples were collected in clot-activator vacutainers and immediately processed for routine blood chemistry reported in Tables S1 and S4. Serum was prepared by blood centrifugation (10 min, 1650 x g, 4°C) and stored at -80°C until lipid extraction and analyses. White adipose tissue biopsies were stored at -80°C until lipid extraction and analysis.

#### Materials for lipid extraction

Synthetic lipid standards [PC 12:0/12:0 (850335), PE 17:0/14:1 (LM-1104), PI 17:0/14:1 (LM-1504), PS 17:0/14:1 (LM-1304), Cer d18:1/17:0 (860517), SM d18:1/12:0 (860583), HexCer d18:1/8:0 (860540)] were from Avanti Polar Lipids Inc. MTBE (methyl-tert-butyl ether) and methylamine (33% in absolute ethanol) were purchased from Sigma Aldrich. Chloroform, methanol, n-butanol and ammonium molybdate were from Acros Organics. LC-MS grade methanol, water and ammonium acetate were from Fluka. HPLC-grade chloroform was purchased from Acros Organics. Monopotassium phosphate, L-ascorbic acid, 70% perchloric acid, hexane, methyl acetate and acetonitrile were from Merck.

#### Serum lipid extraction procedure

Serum lipid extracts were prepared using a modified MTBE extraction protocol with addition of internal lipid standards.^9^^,^^25^ Briefly, 100 μL serum was used, 360 μL methanol and a mix of internal standards were added (400 pmol PC 12:0/12:0, 1000 pmol PE 17:0/14:1, 1000 pmol PI 17:0/14:1, 3300 pmol PS 17:0/14:1, 2500 pmol SM d18:1/12:0, 500 pmol Cer d18:1/17:0 and 100 pmol HexCer d18:1/8:0). After addition of 1.2 mL of MTBE (methyl tert-butyl ether), samples were placed for 10 minutes on a multitube vortexer at 4°C followed by incubation for 1 hour at room temperature (RT) on a shaker. Phase separation was induced by addition of 200 μL MS-grade water. After 10 minutes at RT, samples were centrifuged at 1000 *g* for 10 minutes. The upper (organic) phase was transferred into a 13 mm glass tube and the lower phase was re-extracted with 400 μL artificial upper phase [MTBE/methanol/H_2_O (10:3:1.5, v/v/v)]. The combined organic phases were dried in a vacuum concentrator (CentriVap, Labconco). Lipid extracts derived from MTBE extraction were resuspended in 750 μL chloroform: methanol (1:1), sonicated and vortexed. Resuspended lipids were divided in 3 aliquots. One aliquot was used for glycerophospholipid analysis, a second one for phosphorus assay, and the third aliquot was treated by mild alkaline hydrolysis to enrich for sphingolipids, according to the method by Clarke.^26^ Briefly, 1 mL freshly prepared monomethylamine reagent [methylamine/H_2_O/n-butanol/methanol (5:3:1:4, (v/v/v/v)] was added to the dried lipid extract and then incubated at 53°C for 1 hour in a water bath. Lipids were cooled to RT and then dried. For desalting, the dried lipid extract was resuspended in 300 μL water-saturated n-butanol and then extracted with 150 μL H_2_O. The organic phase was collected, and the aqueous phase was re-extracted twice with 300 μL water-saturated n-butanol. The organic phases were pooled and dried in a vacuum concentrator.

#### SAT lipid extraction procedure

SAT lipid extracts were prepared using a modified 3 Phase Extraction Method^27^ with addition of internal lipid standards. Briefly, 30 mg of tissue were homogenized in N_2_-cold condition (Precellys24 Bertin Instruments) in presence of zirconium oxide beads CK14 (Labgene Scientific) and 200 μL methanol:dichloromethane solution (1:2). After addition of 1 mL of hexane, 1 mL of methyl acetate and 0.75 mL of acetonitrile, samples were vortexed at room temperature and centrifuged at 2000 g for 5 min, resulting in the separation of three distinct phases. The middle layer was re-extracted with 1 mL of hexane and the bottom phase, containing the polar lipids, was collected and dried in a vacuum concentrator. Polar lipid extracts were resuspended in 550 μL chloroforme:methanol (1:1) and divided in 2 aliquots. One aliquot was used for phosphorus assay, and the second one was treated by mild alkaline hydrolysis to enrich for sphingolipids as described above.

#### Determination of total phosphorus

One hundred μL of the total lipid extract, resuspended in chloroform/methanol (1:1), were placed into 13 mm disposable pyrex tubes and dried in a vacuum concentrator. Zero, 2, 5, 10, 20 μL of a 3 mmol/L KH_2_PO_4_ standard solution were placed into separate pyrex tubes. To each tube, distilled water was added to reach 20 μL of aqueous solution. After addition of 140 μL 70% perchloric acid, samples were heated at 180°C for 1 hour in a chemical hood. Then, 800 μL of a freshly prepared solution of water, ammonium molybdate (100 mg/8 mL H_2_O) and ascorbic acid (100 mg/6 mL H_2_O) in a ratio of 5:2:1 (v/v/v) were added. Tubes were heated at 100°C for 5 minutes with a marble on each tube to prevent evaporation. Tubes were cooled at RT for 5 minutes. One hundred μL of each sample was then transferred into a 96-well microplate and the absorbance at 820 nm was measured.

#### Phospho- and sphingolipid analysis by MS

Mass spectrometry analysis for the quantification of phospho- and sphingolipid species was performed using multiple reaction monitoring on a TSQ Vantage Extended Mass Range Mass Spectrometer (ThermoFisher Scientific), equipped with a robotic nanoflow ion source (Triversa Nanomate, Advion Biosciences) as previously described.^9^ Optimized fragmentation was generated using appropriate collision energies and s-lens values for each lipid class. Mass spectrometry data were acquired with TSQ Tune 2.6 SP1 and treated with Xcalibur 4.0 QF2 software (ThermoFisher Scientific). Lipid quantification was carried out using an analysis platform for lipidomics data hosted at EPFL Lausanne Switzerland http://lipidomes.epfl.ch/. Quantification procedure was described in Pietiläinen et al.^28^ Dried lipid extracts were resuspended in 250 μL MS-grade chloroform/methanol (1:1) and further diluted in either chloroform/methanol (1:2) plus 5 mmol/L ammonium acetate (negative ion mode) or in chloroform/methanol/H_2_O (2:7:1) plus 5 mmol/L ammonium acetate (positive ion mode).

### Quantification and statistical analysis

Lipid concentrations were first calculated relative to the relevant internal standards and normalized to the total phosphate content of each total lipid extract for both tissue and serum samples. Lipid concentrations were not corrected for class II isotopic overlaps for the analysis of lipid degree of unsaturation. For comparison of individual lipids or lipid class between different lipids samples, relative lipid concentrations were expressed as a percentage of total lipids detected (mol%). Additional data processing (filtering, normalization, transformation, scaling), statistical analyses, and data plotting were performed using MetaboAnalyst 5.0.^29^ and Prism Graph Pad 8.0. Statistical tests used for comparison between the groups are indicated in the figure legends. Differences were considered significant for p ≤ 0.05 (∗), p ≤ 0.01 (∗∗) and p ≤ 0.001 (∗∗∗). To determine the clustering, k-NN (nearest neighbours with k clusters) was applied for k = 1, 2, and 3 clusters.

### Additional resources

The cohort 1 study was approved by the local ethical commission in Geneva (CCER – 2017-00133). The cohort 2 study had local ethics committee approval from the French Ethical Committee SUD EST IV (Agreement #12/111) and was performed according to the French legislation (Huriet’s law).

## Supplemental information

Document S1. Figures S1 and S2 and Tables S1, S4, and S5

Table S2. Lipidomic data regarding the human serum cohort 1, related to Figures 1 and 2

Table S3. Lipidomic data regarding the human SAT cohort 1, related to Figures 1 and 2
