# Supplementary material for: Alterations of lipid homeostasis in morbid obese patients are partly reversed by bariatric surgery
Source: iScience. 2024 Aug 26;27(9):110820. doi: 10.1016/j.isci.2024.110820 (PMC11409037; doi:10.1016/j.isci.2024.110820)
Supplement: Document S1. Figures S1 and S2 and Tables S1, S4, and S5 [file mmc1.pdf]

iScience, Volume *27*

Supplemental information

Alterations of lipid homeostasis in morbid obese patients are partly reversed by bariatric surgery

Flore Sinturel, Simona Chera, Marie-Claude Brulhart-Meynet, Jonathan Paz Montoya, Etienne Lefai, François R. Jornayvaz, Giovanni D'Angelo, Minoa Karin Jung, Zoltan Pataky, Howard Riezman, and Charna Dibner

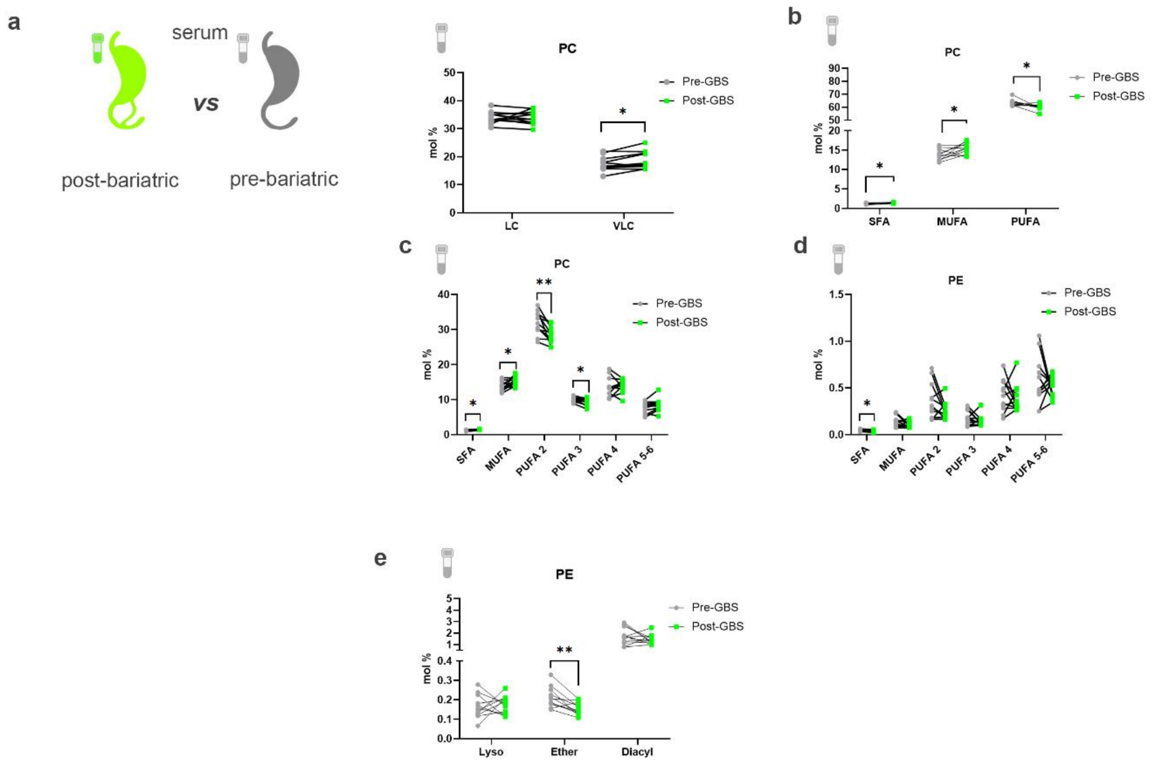

Figure S1: Comparison of differentially abundant serum lipids measured in obese individuals before and after bariatric surgery, Related to Figure 1.

(a) Relative PC level changes (mol%) in sera collected before and after GBS, clustered according to the chain length. LC (long chain C28-34), VLC (very long chain C38-44).

(**b**) Relative PC level changes (mol%) in sera collected before and after GBS, represented according to the class of saturation degree: saturated fatty acids (SFA), monounsaturated fatty acids (MUFA) and polyunsaturated fatty acids (PUFA).

(c-d) Relative PC **(c)** and PE **(d)** level changes (mol%) in sera collected before and after GBS, clustered according to the degree of saturation.

(**e**) Relative PE level changes (mol%) in serum collected before and after GBS, represented according to the nature of the fatty acid linkage (diacyl vs alkyl-acyl (ether) or monoacyl (lyso)).

Statistics for (**c-e**) are paired student's *t*-test. Data for serum (n = 11) pre-GBS and post-GBS are represented as mean ± SEM. * $p < 0.05$; ** $p < 0.01$.

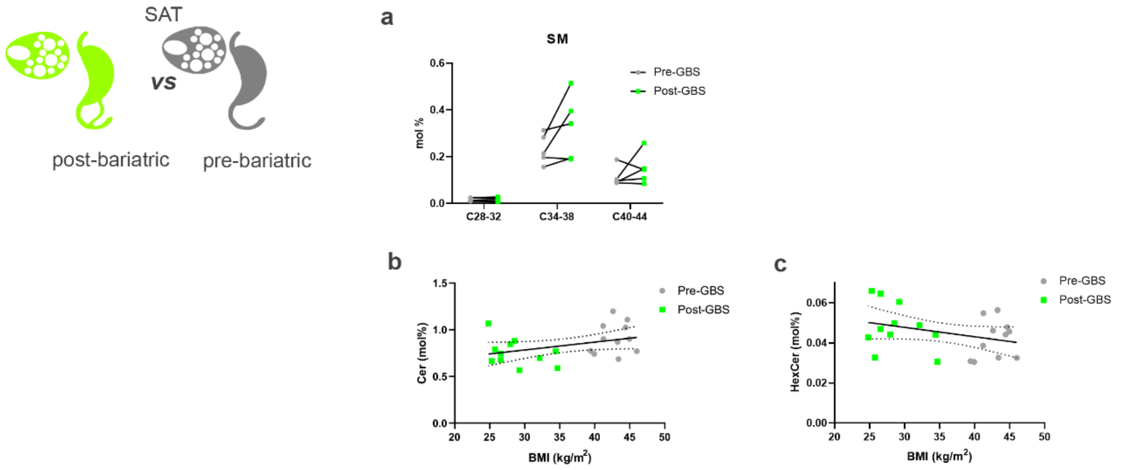

Figure S2: Comparison of differentially abundant SAT lipids measured in obese individuals before and after bariatric surgery, Related to Figure 2.

(**a**) Relative SM level changes (mol%) in sera collected before and after GBS (n = 5), represented according to the chain length. Data are represented as mean ± SEM.

(**b-c**) Association between the relative levels of ceramides (**b**), or HexCer (**c**) detected in sera, and the BMI of the subjects before and after GBS (n = 22, Spearman correlation, for the ceramides R = 0.334, p = 0.1291, for the HexCer, R = -0.237 p = 0.289).

Tables

Table S1. Participant characteristics (cohort 1), Related to STAR Methods.

	Pre GBS (n = 11)			Post GBS (n = 11)			
Serum samples	11			11			
SAT samples	5			5			
Sex	M=4, F=7						
Duration between the 2 samplings (weeks)	65.6 ± 10.41						
**Physical characteristics**			*n*			*n*	*p value*
Age (years)	37.91	± 8.38	11	38.91	± 8.49	11	n/a
BMI (kg. m^{-2})	42.82	± 2.16	11	28.72	± 3.54	11	<**0.001**
Total Body weight (kg)	118.14	± 18.34	11	79.82	± 18.28	11	<**0.001**
Lean body mass (%)	50.85	± 6.08	11	69.99	± 7.70	11	<**0.001**
Fat body mass (%)	49.16	± 6.08	11	29.52	± 7.53	11	<**0.001**
Waist circumference (cm)	121.18	± 10.72	11	95.70	± 14.07	10	**0.002**
Hip circumference (cm)	129.82	± 7.29	11	106.9	± 7.17	10	**0.002**
Waist/hip ratio	0.93	± 0.09	11	0.89	± 0.08	10	0.164
**Laboratory values**							
HbA_{1c} (mmol/mol)	35.5	(38.8-34.4)	5	33.3	(34.4-30.1)	7	0.063
HOMA-IR	5.3	(8.5-4.1)	5	0.9	(4.0-0.4)	7	0.063
Leptin (ng/mL)	122.1	(81.2–153.1)	11	4.3	(3.2–17.2)	11	**0.004**
Adiponectin (ug/mL)	1.3	(3.3-0.4)	8	3.9	(6.5-2.3)	7	0.156
Total Cholesterol (mg/dL)	197	(176–207)	11	144	(117–164)	11	**0.027**
LDL Cholesterol (mg/dL)	124	(108–144)	11	78	(59–87)	11	**0.010**
HDL Cholesterol (mg/dL)	43	(40–47)	11	49	(40–62)	11	0.391
Triglycerides (mg/dL)	98	(64–155)	11	75	(52–102)	11	0.391
Fasting Insulin (mU/L)	22.0	(21.1–28.5)	11	4.1	(1.9–15.2)	11	**0.004**
Fasting glucose (mg/dL)	95.5	(84.7–102.7)	11	87.3	(85.5–105.5)	11	0.848
hs-CRP (mg/l)	8.5	(3.3–11.9)	11	1.5	(0.9–2.6)	11	**0.018**
Resistin (ng/ml)	3.8	(1.9–5.5)	11	9.3	(6.7–12.5)	11	**0.03**
CCL2 (pg/ml)	46.1	(25.0–60.3)	11	15.6	(15.6–15.6)	11	**0.003**
CCL5 (ng/ml)	59.7	(42.2–90.7)	11	22.2	(14.6–27.5)	11	**0.008**

M = male, F= female. LDL: low-density lipoprotein cholesterol. HDL: high-density lipoprotein cholesterol. hs-CRP: high sensitivity C-reactive protein. Normally distributed data are presented as means ± SD; non-normally distributed data are presented as medians (25th - 75th percentile). Group differences were assessed with the Wilcoxon matched-pairs signed-rank test.

Table S4. Participant characteristics (cohort 2), Related to STAR Methods.

	Serum					VAT				
	Control (n = 5)		Obese (n = 16)		*p value*	Control (n = 7)		Obese (n = 11)		*P value*
Sex (M/F)	3/2		6/10			3/4		4/7		
Age (years)	52	(46.50-63.50)	45	(37.00-53.20)	0.1339	51	(40-58)	45	(37-55)	0.675
BMI (kg. m-²)	24.01	(20.12-24.74)	45.34	(42.86-47.24)	**<0.0001**	22.898	(18.94-25.10)	46.65	(44.06-48.28)	**<0.0001**
HbA_{1c} (mmol/mol)	ND		38	(37-38)	n/a	ND		ND		n/a
Fasting glycemia (mmol/L)	ND		*5*	(4.7-5.2)	n/a	ND		ND		n/a

M = male; W = female; visceral adipose tissue (VAT); not determined (ND); Not applicable: (n/a). Data are presented as medians (25th - 75th percentile). Group differences were assessed with the Mann-Whitney *U* test.

Table S5. Comparison of the major lipid classes changes in the patient cohorts 1 and 2, Related to Figures 1-4.

TISSUE	**Post-GBS vs. Pre-GBS**		**Obese vs. Lean**	
	cohort 1		*cohort 2*	
SERUM	**HexCer**<br>**PC (VLC)**<br>**PUFA/MUFA PE**	**Cer**<br>**SM (LC)**<br>**PUFA/MUFA PC**<br>**Ether PE**	**SM**<br>**PC (VLC)**	**PC (LC)**
SAT/VAT	**PI**<br>**HexCer**<br>**SM (LC)**		**PI**<br>**Cer**	**PC**<br>**SM**<br>**HexCer**

Red: increased levels /Green: decreased levels
